# Why do mentally ill patients seek religious places for treatment?

**DOI:** 10.4103/0019-5545.71002

**Published:** 2010

**Authors:** I. Rathinavel, N. R. Prashanth, P. Kasthuri, C.N. Kumar, H. Chandrashekar

**Affiliations:** Department of Psychiatry, Bangalore Medical College and Research Institute, India. E-mail: chanag2061@yahoo.com

Sir,

On the one hand, religious healing serves as an alternative to clinical psychiatric treatment[1] and may help patients cope with schizophrenia or play a part in their treatment adherence.[2] At the other end of the spectrum, lies the shocking Erwadi example, where there was gross human rights violation.[3] Nonetheless, an important proportion (nearly 50%) of patients does attribute the cause of mental illness to super natural forces and seek treatment in religious institutions.[4] This is related to educational level of the caregivers, place of residence and the availability of health care services.[3] A clear understanding of the role of these institutions may have public health implications. Most studies in this area have come from metropolitan cities. We studied, descriptively, the reasons for mentally ill patients visiting Murugamalla, a religious healing center of a village in Karnataka.

Murugamalla is located in Chikballapur district of Karnataka State in South India. Mentally ill are kept there for varying periods of time by the family members. At any point in time, around 50 people reside there. Patients are placed there for various periods of time and then taken back. Few patients, however, stay there for longer term. Family members visit their relatives once in a way. Thirty patients in the center formed the sample for this study (*n*=30). These were the number of patients who were residing when our team visited there for the purpose of data collection. A semi-structured proforma was used to collect the socio-demographic and clinical details. Diagnosis was made according to the International Statistical Classification of Diseases and Related Health Problems 10^th^ Revision (*ICD-10*) criteria. Reasons for visiting that place as told by the patients and their caregivers were collected and tabulated using the SPSS 10.0 version. Data was collected after informed consent from the patient/relatives. This work was cleared by the Institutional Ethics committee.

Mean age of the sample was 32.40 years. The socio-demographic details, diagnoses and reasons for seeking treatment are shown in Tables 1–3. We found that the patients with widely varied diagnoses seek care. Also, the reasons were quite varied. ‘No cure from the hospital/belief that placement will cure the patient’ was the most common reason for placement. This is understandable considering the common beliefs about supernatural causation of mental illnesses.[3] Moreover, religious healing places may indicate a culturally valued refuge for psychiatric patients and they may have a role in providing community mental health care[1] and may indicate the value of a culturally valued refuge for people with severe mental illness. In the background of these findings, it may be reasonable to insist that public health policy makers consider the role of such healing places in proper perspective while planning for mental health care delivery. A limitation of this study was that no structured instruments were used for diagnosis.

**Table 1:** Socio-demographic details

Variable	Number (%)
Gender	
Male	13 (43.3)
Female	17 (56.7)
Locality	
Rural	10 (33.3)
Urban	20 (66.7)
Religion	
Hindu	12 (40)
Muslim	18 (60)
Marital status	
Married	21 (70)
Unmarried	09 (30)
Education	
Illiterate	16 (53.3)
Literate	14 (46.7)
Employment	
Employed	13 (43.3)
Unemployed	17 (56.6)

**Table 2:** Diagnosis

Diagnosis	Number (%)
Schizophrenia	11 (36.7)
Mental retardation	6 (20)
Adjustment disorder	5 (16.7)
Somatoform disorder	02 (6.7)
Depressive disorder	03 (10)
Epilepsy	09 (30)

**Table 3:** Reasons for seeking treatment

Reason	Number (%)*
No cure from the hospital/belief that placement will cure the patient	09 (30)
Nearer to home	06 (20)
Expectation of additional benefit	04 (13)
Feeling that place was safe and secure	04 (13)
Deserted by the family	03 (10)
No cumbersome procedures for admission	03 (10)

* Total percentage exceeds 100% because many patients had more than one reason for seeking treatment
